# Association among helicobacter pylori infection, gastrin level and colorectal cancer in patients aged 50 years and over

**DOI:** 10.12669/pjms.36.5.1993

**Published:** 2020

**Authors:** Chunyan Luan, Zhigang Liu, Yongzhu Li, Tao Dong

**Affiliations:** 1 Dr. Chunyan Luan, Department of Digestion, Affiliated Yidu Central Hospital of Weifang Medical College, Qingzhou, 262500, China; 2 Dr. Zhigang Liu, Department of Cardiology, Affiliated Yidu Central Hospital of Weifang Medical College, Qingzhou, 262500, China; 3 Dr. Yongzhu Li, Department of Digestion, Affiliated Yidu Central Hospital of Weifang Medical College, Qingzhou, 262500, China; 4 Dr. Tao Dong, Department of Digestion, Affiliated Yidu Central Hospital of Weifang Medical College, Qingzhou, 262500, China

**Keywords:** Helicobacter pylori infection, Gastrin, Colorectal cancer, Correlation

## Abstract

**Objectives:**

To study the correlations among helicobacter pylori infection, gastrin and colorectal cancer in patients aged over 50 years old.

**Methods:**

In this study, the patients diagnosed with colorectal cancer treated in the department of digestion of our hospital together with the healthy subjects undergoing colonoscopy for health examination without pathologic findings from August 2016 to July 2019 were enrolled in colorectal cancer or control group. The blood sample was taken in fasting state, and anti-H. pylori IgG and anti-CagA antibodies as well as the level of serum gastrin were measured for all the participants. In addition, the information of each participant including age, gender, obesity, smoking history, alcohol consumption, diabetes mellitus was recorded and analyzed.

**Results:**

Four hundred and twenty-eight patients were enrolled in the colorectal group and 207 healthy subjects were enrolled in the control group. There were not significant differences in the positive rate of Ig G and Cag A and family history between the two groups (p>0.05), but there were significant differences in gastrin level, obesity, smoking history, alcohol consumption and diabetes mellitus between the two groups (p<0.05). In addition, the multivariable analysis showed that obesity, smoking history, alcoholism and diabetes mellitus have the strongest influence on the formation of colorectal cancer, while the level of gastrin didn’t show the influence.

**Conclusions:**

No significant correlations among H. pylori infection, the level of gastrin, and the occurrence of CRC in patients with a minimum age of 50 years, suggesting elder colorectal cancer patients may have a different carcinogenic mechanism from those younger patients.

## INTRODUCTION

Colorectal cancer (CRC) is one of the most common digestive malignancies in the world, and rapid increases of CRC incidence and mortality are observed in many countries.^1^ It was reported that 90% of new cases and over 90% of deaths of CRC occurred at 50 years and beyond.^2^ As a result, it is critical to study the mechanism of CRC in order to prevent the fatal disease, especially in patients aged over 50 years old.

In recent decades, numerous studies have elucidated the potential association of helicobacter pylori (H. pylori) infection with CRC,^3^,^4^ and H. pylori infection is regarded as an important cause for CRC.^5^ Meanwhile, as the incidence and mortality rate of CRC increase sharply with age, some scholars tried to study the association between H. pylori infection and the occurrence of CRC in patients aged over 50 years old. In a study of 377 colorectal cancer patients with a minimum age of 50 years, Selgrad and colleagues found H. pylori infection is associated with an increased risk for the development of colonic neoplasm.^6^ However, in another study carried out by Park and colleagues, they found H. pylori infection independently increased the risk of advanced colorectal neoplasia in patients aged <50 years but not in patients aged ≥50 years.^7^ Similarly, in another study of 392 elderly participants and 774 controls, Blase and colleagues didn’t find an association between H. pylori infection and colorectal cancer risk.^8^ Obviously, the conclusions were controversial, and it need further study to determine if there is a close correlation between H. pylori infection and colorectal cancer occurrence.

As to the carcinogenic mechanisms of H. pylori infection, the viewpoint of the increasing of gastrin level was highlighted.^3^ A number of studies advocated there was a positive association between higher serum gastrin level and risk of colorectal cancer,^8^ in which the reduced gastric acid secretion resulting from increased gastrin level and H. pylori infection promotes the changes in colorectal microflora to lead to colorectal carcinogenesis.^9^ However, different viewpoints have been published in recent years, Selgrad found, in a study which only recruited the colorectal cancer patients aged more than 50 years old, that hypergastrinemia didn’t increase the risk of any colonic neoplasms.^6^ As a result, we think a clinical study is worth performing to determine the exact association between H. pylori infection, gastrin level and CRC occurrence in patients aged over 50 years old, which may clarify if these CRC patients have a different carcinogenic mechanism. Nevertheless, up to now, few such studies have been carried out, and the issue is still not clear.

Therefore, in the current study, we analyzed the patients diagnosed with CRC treated in our hospital and some healthy subjects during the period between August 2016 and July 2019, and our objective was to detect the correlations among H. pylori infection, the level of gastrin, and the occurrence of CRC in patients aged more than 50 years old, and determine if CRC patients aged over 50 years have a different carcinogenic mechanism. The study may help physicians and patients better recognize the fatal disease.

## METHODS

In this study, the patients diagnosed with colorectal cancer treated in the department of digestion of our hospital from August 2016 to July 2019 were analyzed. The inclusion criteria were: 1) patients newly diagnosed with colorectal cancer based on colonoscopy and histological examination of tumor biopsies;^10^ 2) patients with a minimum age of 50 years; 3) patients agreed to participate the study. To facilitate the study, those patients with inflammatory bowel disease, nonadenomatous polyps, and history of cancer or eradication therapy of helicobacter pylori infection prior to colonoscopy was excluded from the study.^11^ In addition, the healthy individuals undergoing colonoscopy for health examination without pathologic findings, such as polyps, neoplasms, or inflammatory diseases, were enrolled as control group. The study was approved by the Ethical Review Board of our hospital (Date: November 20, 2019), and a written informed consent was signed by all participants.

The information of the patients and healthy subjects such as age, gender, and those possible confounders evaluated known risk factors for CRC, including obesity (defined as BMI > 30), smoking history (ever/never), alcohol consumption (alcoholism), diabetes mellitus were recorded and compared. In addition, about 5-7 ml blood sample was taken from all the included participants in fasting state. Anti-H. pylori IgG and anti-CagA antibodies were measured using an H. pylori IgG enzyme-linked immunosorbent assay and a CagA IgG kit, respectively, based on manufacturers’ instructions.^6^ According to the presence of H. pylori-specific IgG greater than or equal to 30 enzyme immunounits and/or the presence of anti- CagA IgG greater than or equal to 6.25 U/ml,^6^ the patients were regarded H. pylori positive, whereas the lack of both antibodies were regarded H. pylori negative. The level of serum gastrin was tested in all participants and as described by the manufacturer.

The statistics was conducted using SPSS 21.0 (SPSS Inc., Chicago, IL, United States). Measurement data were presented as mean ± standard deviation and compared using the Student’s *t* test. The categorical variables were compared using Chi-squared test, and correlational analyses were performed using multivariable logistic regression analysis. A p value less than 0.05 was regarded as statistical significance.

## RESULTS

In the current study, 428 patients were enrolled in the colorectal group and 207 healthy subjects in control group. In colorectal cancer group, there were 249 males and 179 females, aged from 50 years to 78 years, and in the control group, there were 116 males and 91 females, aged from 50 years to 79 years. There were no significant differences in age and gender between the two groups, demonstrating that the colorectal cancer patients and controls were well matched regarding gender and age. The clinical characteristics of the two groups are shown in Table-I.

**Table-I:** Clinical characteristics of the two groups.

	Colorectal cancer (n=428)	Control group (n=207)	p value
Gender			>0.05
Male	249	116	
Female	179	91	
Age (year)	58.6±7.9	56.6±8.5	>0.05
Ig G(+)	218	93	>0.05
Cag A(+)	235	109	>0.05
Gastrin (>38.8Pm/L)	232	19	<0.0001
Obesity (BMI > 30), N (%)	158	49	<0.001
Smoking (ever), N (%)	202	72	<0.05
Alcoholism, N (%)	28	4	<0.05
Diabetes mellitus, N (%)	93	25	<0.01
Family history (yes/no)	59/369	32/175	>0.05

In colorectal cancer group, 218 and 235 patients were positive in Ig G (+) and Cag A (+), the rate was 50.9% and 54.9%, and in the control group, 93 and 109 patients were positive in Ig G (+) and Cag A (+), and the rate was 44.9% and 52.7%, respectively. In colorectal cancer group, 59 patients had family history of CRC, and in the control group, 32 had family history. There were not significant differences in the positive rate of Ig G and Cag A as well as family history between the two groups (p>0.05) (Table-I).

**Fig.1:**
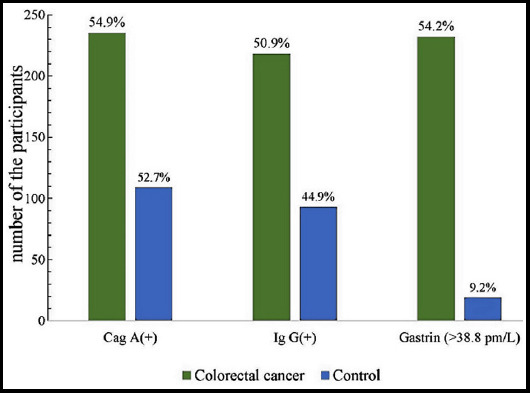
The distribution of the patients with Cag A (+), Ig (+) and the level of gastrin higher than 38.8pm/L in the two groups.

However, the number of the patients with the level of gastrin higher than 38.8 pm/L was 232 in in colorectal cancer group, and 19 in the control group, there was a significant difference between the two groups (P<0.05) (Fig.1). Similarly, in terms of obesity, smoking history, alcohol consumption (alcoholism), and diabetes mellitus, significant differences were found between the two groups (p<0.05).

In addition, the multivariable logistic regression analysis indicated that, regardless of OR values for individual variables, the confounders including obesity, smoking history, alcoholism and diabetes mellitus results showed OR over 1.0 and have the strongest influence on the formation of colorectal cancer, while the level of gastrin didn’t show the influence (Table-II).

**Table-II:** Multivariable analysis with logistic regression of variables in both groups.

Variables	Regression coefficient	Odds ratio
Dependent variable		
Colorectal cancer=1		
Control=0		
Independent variable		
Gastrin (<38.89)=0	3.45	0.85
Gastrin (>38.89)=1		
Obesity(BMI > 30)		3.17*
No=0	5.16	
Yes=1		
Smoking (ever)		2.74*
No=0	-3.28	
Yes=1		
Alcoholism		4.38*
No=0	-0.39	
Yes=1		
Diabetes mellitus		0.96
No=0	-2.16	
Yes=1		

***Note:***

* indicates a significant difference.

## DISCUSSION

In this study, we tried to detect the associations among H. pylori infection, the level of gastrin, and the occurrence of CRC in patients aged more than 50 years old, and determine if CRC patients aged over 50 years old have a different carcinogenic mechanism. To the best of our knowledge, few studies has been carried out in this regard.

Many studies have demonstrated the potential association between helicobacter pylori infection with CRC.^3^,^12^ However, in the current study, we found the positive rate of Ig G (+) and Cag A (+) was 50.9% and 54.9% in colorectal cancer group, and the rate in the control group was 44.9% and 52.7%, there was no significant difference in each positive rate between the two groups. In this study, only patients and healthy subjects aged more than 50 years were enrolled, and the results indicate no potential association between helicobacter pylori infection and CRC, and elucidate that in colorectal cancer patients aged greater than 50 years old H. pylori infection may not be a crucial factor to influence the occurrence of CRC. Our result is consistent with the studies from Park^7^ and Blasé.^8^

A number of studies have evaluated the correlation between obesity, smoking history, alcohol consumption, diabetes mellitus and CRC. In a study of 918 CRC cases and 1,021 controls, Boyle found that alcohol, smoking, and diabetes were associated with an increased risk of colorectal cancer.^13^ In another study, Grosso reported the same conclusion.^14^ In the current study, our conclusion was consistent with these studies. However, in terms of gastrin level, although a significant difference was detected between the two groups, the multivariable analysis showed it didn’t have a strongest influence on the formation of colorectal cancer. The results indicate that although gastrin may take part in the occurrence of CRC, it may not be the crucial factor to influence the progression. Some various factors, including obesity, smoking history, alcohol consumption, diabetes mellitus or other factors may play a more crucial role in the carcinogenic mechanism of CRC patients aged more than 50 years old.

According to the mechanism of CRC development, many scholars advocates the viewpoints that a possible causal link between H. pylori infection, gastrin and colorectal cancer. Persistent H. pylori exposure elicits high gastrin level, which is a putative trophic factor for the colorectal mucosa and thereby a promoter of mutagenesis. In addition, hypergastrinemia has been shown to be directly mitogenic on either normal or neoplastic colonic cells.^9^ However, in this study we got different results, and didn’t find significant correlations among H. pylori infection, the level of gastrin, and the occurrence of CRC in patients with a minimum age of 50 years, and the current study suggests elder CRC patients may have a different carcinogenic mechanism from those younger patients.

### Limitations of the study

First, we advocated a different carcinogenic mechanism in colorectal cancer patients aged over 50 years old, but the mechanism is still unclear, as we didn’t focus on the pathological details in this study. Second, some of our results were consistent with those published studies, but some were still inconsistent. For an instance, in a study carried out by Fernandez de Larrea-Baz and colleagues, they found gender may also influence the occurrence of CRC, but we didn’t find the same association.^15^ Third, due to the multifactorial nature of CRC, other confounders including dietary habit, living environment, and drug use, may also affect the development of CRC, but some of these epidemiological associations are difficult to draw, so they were not considered in this study. Hence, more studies should be performed in the future to further clarify these issues.

### Authors’ Contribution

**CYL:** Conceived, designed and did statistical analysis,

**CYL, YZL, TD & ZGL:** Did data collection and manuscript writing,

**CYL, ZGL:** Did review and final approval of manuscript.

**CYL** was responsible for the accuracy or integrity of the work.
